# Optimising data visualisation formats for reporting medication-related care quality indicators: residential aged care staff preferences, interpretations and actions

**DOI:** 10.1136/bmjoq-2025-003897

**Published:** 2026-09-08

**Authors:** Isabelle Carmen Meulenbroeks, Rachel Urwin, Andrea Timothy, Ben Newell, Tracy Bucknall, Sangita Neupane, Tim Badgery-Parker, Karla Seaman, Johanna I Westbrook

**Affiliations:** 1 Australian Institute of Health Innovation, Macquarie University, Sydney, New South Wales, Australia; 2 School of Psychology, University of New South Wales, Sydney, New South Wales, Australia; 3 Institute of Climate Risk & Response, University of New South Wales, Sydney, New South Wales, Australia; 4 Centre for Quality and Patient Safety Research, Deakin University, Melbourne, Victoria, Australia; 5 Alfred Health, Melbourne, Victoria, Australia

**Keywords:** Nursing homes, Continuous quality improvement, Surveys, Quality improvement

## Abstract

**Introduction:**

Data visualisations are widely used to communicate complex performance information within healthcare quality improvement feedback cycles. When tailored to their intended audience, these visualisations have the potential to stimulate change to improve care quality. This study assessed which data visualisation formats Australian residential aged care (RAC) staff interpret correctly, prefer and find informative enough to prompt action to improve care quality.

**Methods:**

A 37-item survey was administered via REDCap to Australian RAC staff between September and November 2024. The survey included demographic and data visualisation questions. Participants evaluated five cross-sectional data visualisation formats—table, star rating, bar chart, funnel plot and caterpillar plot—and two longitudinal data visualisation formats—box plot and line graph—based on interpretability, preference and actionability. Quantitative data were analysed descriptively, while qualitative responses underwent content analysis.

**Results:**

42 RAC staff participated in the survey. Respondents favoured bar charts for reporting cross-sectional data (n=23, 55%) with 79% (n=33) interpreting them correctly. For longitudinal data, 30 respondents (91%) favoured line graphs, with 71% (n=24) interpreting them accurately. Star ratings were the least preferred cross-sectional data visualisation format due to insufficient contextual information. In open-ended responses, respondents suggested that data visualisations would prompt them to review outliers, conduct medication reviews and educate staff. However, some respondents indicated that more contextual information, such as facility size and resident characteristics, was required in visualisations before they would take action to improve care quality.

**Conclusion:**

Bar charts and line graphs appeared to be the most suitable visualisation formats to convey medication-related care quality information to RAC staff for cross-sectional and longitudinal data respectively, as they were generally interpreted correctly with relative ease, were perceived as actionable and were preferred by most participants. In contrast, complex visualisations, such as funnel and box plots, were not preferred and were misinterpreted by half or more of the participants.

**WHAT IS ALREADY KNOWN ON THIS TOPIC**

- Tailoring data visualisations to the information needs and data literacy of health professionals can effectively communicate care quality performance and prompt staff to improve underperforming areas. However, little is known about what type of data visualisation formats are best for RAC staff.

**WHAT THIS STUDY ADDS**

- This study addressed that gap by identifying bar charts and line graphs as the most suitable formats for presenting medication-related care quality data to RAC staff. These formats appeared to be more easily interpreted, were preferred by participants and were more often reported as prompting intended action.

**HOW THIS STUDY MIGHT AFFECT RESEARCH, PRACTICE OR POLICY**

- The results suggest that data visualisations in RAC should strike a balance, avoiding overly simplistic formats like star ratings, which lack sufficient detail, as well as overly complex formats, which may reduce usability.

## Introduction

 Data visualisations such as bar charts, line graphs and pictorials are powerful tools for communicating complex information within healthcare quality-improvement feedback cycles. A feedback cycle is a structured process in which performance data are collected, presented, used to inform decisions and implement change and then re-measured to assess impact, creating iterative cycles of learning and improvement.^1 2^ This cyclical structure underpins established quality-improvement methods, including learning health systems, Six Sigma and Plan–Do–Study–Act.^3–5^ When clear and targeted data visualisations are used within healthcare feedback cycles they can guide decision-making and prompt action to improve care quality.^1 6^ For example, in US hospitals, data visualisations of heart failure readmission rates prompted hospital staff to implement targeted interventions in high-risk groups, reducing readmissions.^7^ Consumer-facing visualisations have also influenced care quality; a regularly updated US star-rating system for healthcare facility performance led to increased consumer selection of higher-performing providers and a 6% increase in their market share.^8^ Medication-related care has similarly benefited from ongoing data visualisation: in the US prescriber peer-comparison graphs have resulted in a reduction in opioid prescribing^9^ and an increase in venous thromboembolism prophylaxis prescription after surgery.^10^

Improving care quality in residential aged care (RAC) homes, also known as long-term care or nursing homes, is an urgent international priority. Decades of inquiries in countries, such as Australia, the UK and the US have documented serious and recurrent failures in care delivery, including neglect, abuse, inadequate staffing, underservicing, low infection control standards and inappropriate medication use.^11–15^ To drive care quality improvement in RAC, health systems internationally—including those in Australia, Canada and the US—have introduced audit and feedback cycles to routinely collect and publicly report key indicators of care quality, such as falls, pressure injuries, staffing levels and polypharmacy.^16–18^ Within this context, our research team established the Australian National Aged Care Medication Roundtable in 2022 to support quality improvement in Australian RAC, with a specific focus on medication safety.^19^ The Roundtable brings together aged care providers, policymakers and consumer representatives and uses data-driven feedback cycles to support improvement. In the Roundtable, facility-level benchmarking reports are produced quarterly, discussed collectively and used to design and implement interventions that are reassessed.

Despite the growing reliance on performance data to drive improvement in RAC settings internationally, little is known about which data visualisation formats best support interpretation, decision-making and action among RAC staff.^20^ Evidence from acute and primary care settings suggests that hospital and community care professionals prefer and accurately interpret formats such as bar charts and symbol-based tables.^21–23^ It is not known whether these findings are applicable to RAC settings, as the sector faces unique challenges including limited statistical training among staff and a historically low emphasis on quality improvement.^11 23 24^ To address this knowledge gap, we conducted a survey of RAC staff to identify which data visualisation formats staff find easiest to interpret correctly, are most likely to prompt action to improve care quality and most preferred for regular use. The findings will enable the development of evidence-based, sector-tailored care quality reports for the National Aged Care Medication Roundtable^19^ and are likely to be relevant internationally given the limited evidence on data visualisation literacy in the aged care sector.

## Methods

### Survey design

A 37-item survey, including multiple choice and open-ended questions, was developed by the research team drawing on previous mixed-method research, which explored how healthcare staff interpret and action data.^25–27^ The survey examined data visualisation preferences, interpretation accuracy and intended actions among aged care staff, reflecting the sequential steps required for feedback cycles to translate performance data into quality improvement.

The survey was piloted among the research team, which included researchers who have experience working in RAC, and then refined in response to feedback. The final survey received ethics approval from the Macquarie University Human Research Ethics Committee (Project ID: 11267). The survey was administered through REDCap, hosted by Macquarie University.^28 29^ A copy of the survey is available in online supplemental material 1.

Five items collected details about respondents and their work in RAC: job role (eg, nurse, care manager), years of experience (<1, 1–4, 5–9,>10 years’ experience), type of RAC facility respondent is employed at (government or private facility), day-to-day use of quality indicator data (5-point Likert scale from ‘never’ to ‘always’) and core tasks performed in their current role. Respondents were provided with a list of 10 tasks derived from previous surveys of RAC staff^30^ and could select multiple items and provide an open-text response.

**Figure 1:**
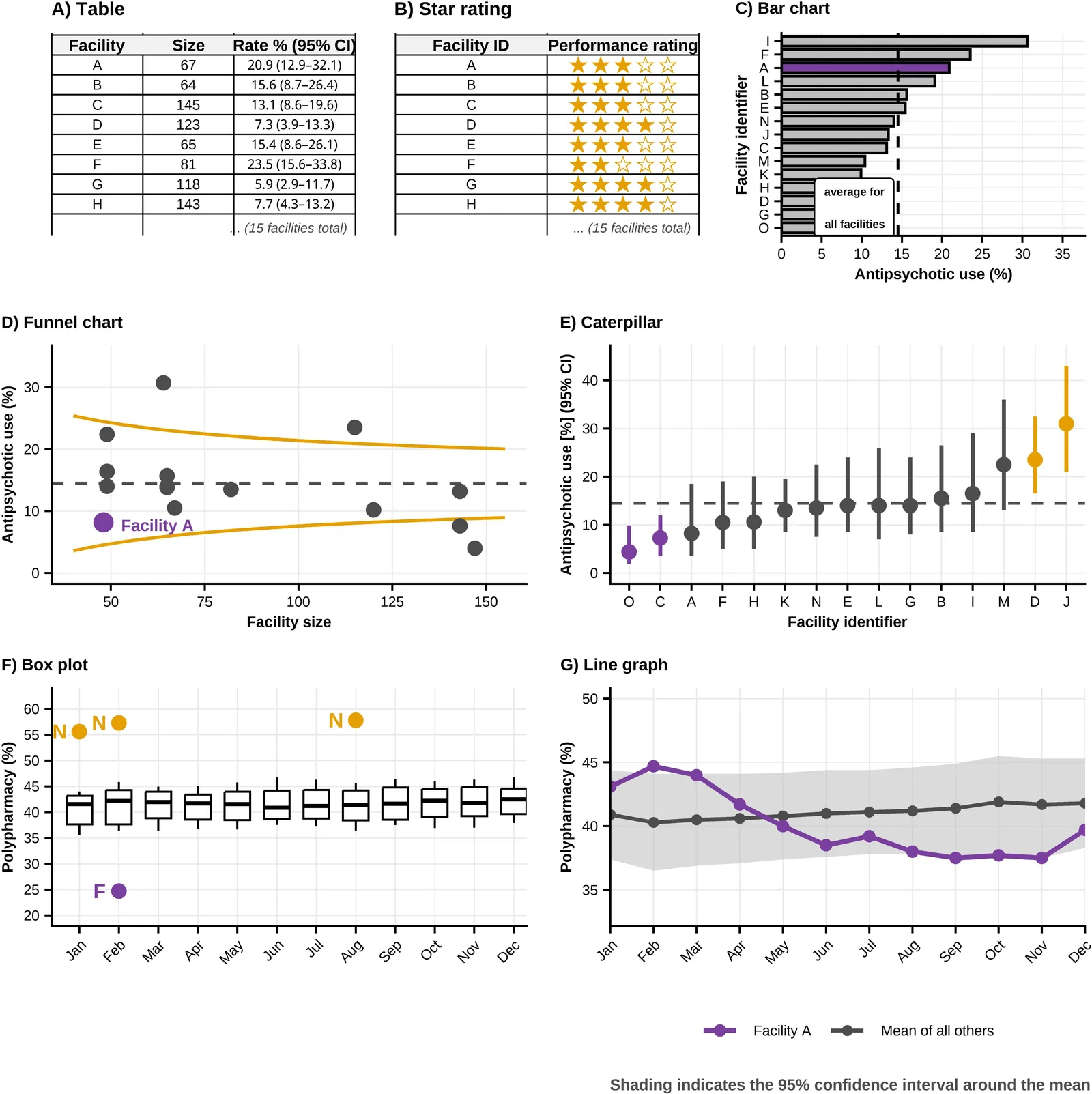
Data visualisation formats Case 1: Cross-sectional data on antipsychotic use in 15 RAC facilities: A) Table of 15 facilities (labelled A–O) showing facility size, proportion of residents receiving antipsychotics and 95% CIs. (**B**) Five-star rating assigned to each facility, where a higher number of stars indicates lower antipsychotic use. (**C**) Bar chart depicting the proportion of residents receiving antipsychotics per facility, with a median line indicating overall cohort performance. (**D**) Funnel plot displaying facility-level antipsychotic use relative to facility size, with control limits included. (**E**) Caterpillar plot ranking facilities by mean antipsychotic use, with a cohort mean line and 95% CIs for each facility. Case 2: Longitudinal data on polypharmacy in 15 RAC facilities: F) Box plot showing median polypharmacy percentages over time for all facilities, including IQRs and outliers. (**G**) Line graph comparing a single facility’s polypharmacy trajectory with the cohort mean, with CIs indicated by shading. RAC, residential aged care.

Seven data visualisation formats (table, star rating, bar chart, funnel plot, caterpillar plot, line graph and box plot) using mock medication use data were presented to participants in two cases. The first case presented cross-sectional data on antipsychotic use; the second case longitudinal data on polypharmacy (figure 1). Tables, bar charts, line graphs and box, funnel and caterpillar plots all displayed numeric summary data. In the five-star rating system presented, pictorials were used to convey that high-quality care (five stars) corresponded to low rates of antipsychotic use.

To evaluate participants’ comprehension of each data format, the survey included multiple-choice questions. For example, after viewing the bar chart, participants were asked “What is the average antipsychotic use across facilities?” with response options: A) 17.5%, B) 10%, C) 23%, D) 30.5%, E) not sure. In addition, participants were also asked to rate the ease of interpreting each format using a 5-point Likert scale from ‘very difficult’ to ‘very easy’. They were also asked whether the data visualisation format provided sufficient information to support action aimed at improving care quality (yes/no/unsure). If they answered ‘yes’ they had the option to elaborate in an open-text field and to specify what actions they would take. In this study ‘action’ and ‘actionability’ are used to refer to data-guided behaviour, where the participant reports that the data presented would prompt them to explore or modify care practices.

After each case, participants were asked to indicate their most and least preferred data visualisation formats (multiple choice). Following Case 1, participants chose their preferred format between a table, star rating, bar chart, funnel plot, and caterpillar plot. After Case 2, respondents were asked whether they preferred a box plot or line graph. Questions exploring participant preferences for graph visualisation were based on similar studies which explored correct interpretation of graphs and preferred graph types in cancer patients.^31^

### Recruitment

RAC care staff (eg, personal care workers), nurses, pharmacists, other allied health professionals, care/facility managers and aged care policy workers working in the RAC sector were eligible to participate in the survey. Participants were recruited between 9 September 2024 and 17 November 2024, and were offered a 1 in 10 chance to win a AU$50 gift voucher for completing the survey. Participants were recruited primarily through partner aged care organisations and providers (46 RAC homes). Additional recruitment material was distributed through peak and professional body newsletters and forums, contact lists from past research activities and through convenience networks. Participants who started the survey in REDCap but did not complete at least Case 1 were excluded from analyses.

### Analysis

Quantitative data were analysed using Stata, while qualitative data were organised in Excel.^32 33^ Questions assessing participants’ understanding of the data visualisation formats were recoded as binary variables, indicating either a correct or incorrect interpretation. Qualitative and quantitative data were analysed and are presented separately.

Quantitative survey responses, including participant demographics, were descriptively analysed using means, medians and ranges. Fisher’s exact test was used to investigate whether there were demographic differences between people who dropped out after completing demographic questions and therefore were not included in the analysis and participants who completed the entire survey. Statistical significance was set at a p<0.05.

Content analysis of the qualitative data was conducted. Open-text responses were first inductively coded to identify key phrases and then recontextualised in relation to the survey questions. The conceptualised responses were then recoded into categories to answer the research question. Coding was undertaken by one author but the process was routinely checked and revised when appropriate by the research team.

## Results

### Participant demographics

Eighty people started the survey; 42 respondents completed at least Case 1 (cross-sectional data for antipsychotic use), and their data were retained for analysis. There was no difference in the demographic details between respondents who completed only the demographic questions (whose data were excluded; n=38) and those who completed at least Case 1 (n=42) (online supplemental tables 1-3). 33 participants completed Case 2.

Respondents were predominantly personal care workers (n=12) and registered nurses (n=10). Participants had a wide range of experience in the aged care sector: 19% had worked less than 1 year and 35% had more than 10 years. Most participants reported that they either always (46%) or often (25%) encountered quality indicator data relating to medication management in their role in the RAC sector (table 1). In their role, many participants indicated they provided leadership and oversight (48%). Additionally, 43% engaged in implementing strategies aimed at improving care and 43% investigated the care needs of residents.

**Table 1:** Participant characteristics (n=42)

Years of experience in aged care sector	
< 1 year	8 (19%)
1-4 years	10 (24%)
5-9 years	9 (21%)
10 or more years	15 (36%)
Role	
Personal care worker	12 (29%)
Registered nurse	10 (24%)
Allied health practitioner	2 (5%)
General practitioner	1 (2%)
Policy advisor	2 (5%)
Residential/facility manager	1 (2%)
Care manager	3 (7%)
Pharmacist	6 (14%)
Other*	5 (12%)
In your current role, do you encounter or use quality indicator data relating to medication management	
Never	5 (13%)
Rarely	2 (5%)
Sometimes	4 (10%)
Often	10 (26%)
Always	18 (46%)
Data roles performed	
Provide governance, leadership and oversight	20 (48%)
Ensure there are adequate resources (eg, equipment, materials, appropriately trained staff etc.) to support quality improvement activities	17 (41%)
Review performance and set targets to improve quality of care	13 (31%)
Investigate and implement interventions that can improve care	18 (43%)
Personalise and discuss quality indicator data so it is relevant and meaningful to staff	12 (29%)
Collect, report and respond to quality indicators	17 (41%)
Check the reliability and accuracy of collected quality indicator data	9 (21%)
Report quality indicator information to resident, and their families	5 (12%)
Investigate if there are changes in the care needs of residents (eg, more complex needs, and make appropriate adaptations to improve care	18 (43%)
Partner with other organisations or facilities to share information to improve care	8 (19%)
Other†	5 (12%)

* 4 Managers, 1 Consultant.

† 1 Review policy, 4 Care tasks.

### Quantitative results: Data visualisation interpretation, ease of interpretation, preferences and actionability

#### Case 1: Cross-sectional data visualisation formats

Most participants found bar charts easy to interpret (median response: easy; table 2). The perceived ease was reflected in accurate interpretation with 79% of respondents correctly answering two questions based on the bar charts. Preferences aligned with performance; 55% of respondents chose bar charts over other formats including tables, funnel plots, caterpillar plots and star ratings.

**Table 2:** Number of participants who answered interpretation questions correctly, for each data format and their most and least preferred data format

	Participants who interpreted visualisations correctly (n (%) (number of questions))	Perceived ease of interpretation* (median, IQR)	Most preferred data format	Least preferred data format†
Cross-sectional data formats				
Bar chart	33 (79%)^2^	Easy (4, 3**–**5)	23 (55%)	2 (5%)
Table	34 (81%)^1^	Neutral (3, 2**–**4)	7 (17%)	8 (20%)
Funnel plot	17 (40%)^3^	Neutral (3, 2**–**4)	2 (5%)	7 (17%)
Caterpillar plot	16 (38%)^2^	Neutral (3, 2**–**4)	4 (10%)	5 (12%)
Star rating	28 (67%)^2^	Neutral (3, 2**–**4)	6 (14%)	19 (46%)
Longitudinal data formats				
Box plot	18 (53%)^2^	Difficult (2, 1**–**3)	3 (9%)	N/A
Line graph	24 (71%)^2^	Neutral (3, 3**–**4)	30 (91%)	N/A

* Interpretation: the Likert category corresponding to the median value: 1, Very difficult; 2, Difficult; 3, Neutral; 4, Easy; 5, Very easy.

† A 41/42 participants completed least preferred cross-sectional data format question

Participants expressed neutral views about the perceived ease of interpretation of the alternative cross-sectional data visualisation formats presented in Case 1, namely tables, funnel plots, caterpillar plots and star ratings. However, in practice, these neutral views did not translate into accurate interpretation. Fewer than half of the participants correctly answered interpretation questions for funnel plots (40%) and caterpillar plots (38%). Interestingly, while 67% of participants interpreted star ratings correctly, this format was still ranked as the least preferred among all cross-sectional data visualisations in Case 1.

#### Case 2: Longitudinal data visualisation formats

Participants demonstrated greater accuracy in interpretation with line graphs compared with box plots, with 71% of participants answering two questions about line graphs correctly, versus 53% for box plots (table 2). This performance was consistent with perceived ease of interpretation with line graphs rated neutral in terms of ease of interpretation, while box plots were rated as difficult to interpret on a five-point Likert scale. Line graphs were also the preferred data visualisation format, with 91% of participants selecting them as their preferred data visualisation format for longitudinal data over box plots.

#### Actionability

Participants were asked whether each data visualisation provided sufficient information to prompt intended action on medication-related care quality in RAC, recognising that responses reflect perceived rather than observed action. Overall, responses were mixed, with respondents divided across ‘yes’, ‘no’ and ‘unsure’, with no clear patterns emerging. In Case 1, focused on cross-sectional data, bar charts were considered the most informative, with 44% of respondents indicating they provided enough detail to take action.

Star ratings were considered the least informative format for stimulating action on medication-related care quality, with 37% of participants reporting 'no’—meaning the star rating did not provide enough information—compared with 17% for tables. Funnel plots generated the most uncertainty with 42% of participants unsure whether they contained enough information to support action, despite presenting additional context such as facility size as part of the visualisation.

In Case 2, which presented longitudinal data, box plots received the highest level of uncertainty about whether they could inform intent to act on the data, with 50% of participants unsure. Line graphs were the most actionable, with 50% reporting they contained all the necessary information to act (table 3).

**Table 3:** Does this visualisation provide you with enough information to initiate action?

	Response		
**Visualisation type**	**Yes**	**No**	**Not sure**
Cross-sectional			
Bar chart	18 (43%)	10 (24%)	14 (33%)
Table	18 (44%)	7 (17%)	16 (39%)
Funnel	13 (33%)	10 (25%)	17 (43%)
Caterpillar	15 (36%)	11 (26%)	16 (38%)
Star rating	13 (32%)	15 (37%)	13 (32%)
Longitudinal			
Boxplot	9 (27%)	8 (24%)	17 (50%)
Line graph	17 (50%)	6 (18%)	11 (32%)

Survey participants were asked whether a specific facility’s performance, presented through different data visualisation formats as better than average, average or worse than average, warranted further investigation or action (table 4). In the cases presented, most participants indicated they would investigate or act to improve care for facilities performing worse than average, with 88% for bar charts and 86% for tables. For average-performing facilities, participants generally reported no action was needed, ranging from 50% for box plots to 61% for funnel plots. Similarly, when facilities performed better than average, the majority indicated no action was required, with 57% of respondents expressing this view in response to star ratings.

**Table 4:** Facility performance and need to respond

Performance of highlighted facility	Data visualisation format	In this facility the rate of medication use…			
		May indicate inappropriate use of medicines. further investigation/action may be needed	Indicates that no action is required	Not sure	Other
Worse than average	Bar chart	37 (88%)	1 (2%)	3 (7%)	1 (2%)
	Table	36 (86%)	0 (0%)	6 (14%)	0 (0%)
Within average range	Funnel plot	4 (10%)	25 (61%)	12 (29%)	0 (0%)
	Caterpillar	5 (12%)	25 (60%)	12 (29%)	0 (0%)
	Box plot	7 (21%)	17 (50%)	10 (29%)	0 (0%)
	Line graph	9 (27%)	18 (53%)	5 (15%)	2 (6%)
Better than average	Star rating	5 (12%)	24 (57%)	13 (31%)	0 (0%)

### Qualitative results: actions taken and information needs

Participants were asked to provide open-text feedback on whether the data visualisation would prompt action (online supplemental table 4). Among those who responded ‘yes’, many indicated they would investigate care quality in facilities that appeared to be performing poorly. For example, one participant noted they would *“determine appropriate levels of prescription and review the cohort at the facility”* to understand whether excessive antipsychotic use was appropriate for the case mix at the facility (Participant 24, Data visualisation format: Bar chart). Less common actions included conducting or organising individual medication reviews, discussing findings in clinical meetings and providing staff education. For example, *“Provide education to facilities above average usage”* (Participant 68, Data visualisation format: Table) and suggesting a review by a psychogeriatrician (Participant 42, Data visualisation format: Table).

Participants who responded 'no’—that the data visualisation did not provide enough information to act on—often cited the need for more context, such as the proportion of residents with psychological conditions, facility size or clearer presentations of data. Examples included: *“I cannot determine as I could not understand the graph”* (Participant 18, Data visualisation format: Boxplot), *“No context, no adjustments”* (Participant 8, Data visualisation format: Star rating), and *“I need to know about the patient population in each facility… there may be larger cohorts in whom antipsychotic use is entirely appropriate”* (Participant 100, Data visualisation format: Table).

## Discussion

Our study found that bar charts and line graphs were the most suitable visualisation formats for presenting cross-sectional and longitudinal medication-related data, respectively, to RAC staff. This was based on participants’ abilities to interpret data correctly, ease of interpretation, their stated preferences, and whether the format was reported to prompt intended action. Tables may also be an appropriate format for cross-sectional data as they were interpreted accurately more often than bar charts (81% vs 79%); however, they were less preferred.

The results demonstrate that data visualisations which present simple summary statistics, such as tables and bar charts for cross-sectional data and line graphs for longitudinal data, were correctly interpreted by around 80% of participants, whereas more complex formats, including funnel and caterpillar plots with CIs and facility size, were misinterpreted by over half of the participants. These findings mirror those reported in other healthcare contexts: studies with home care nurses found that 80% interpreted bar charts, tables and line graphs correctly, while only 40% could interpret more complex diagrams such as spider plots.^24^ Similarly, healthcare decision-makers often struggle to comprehend funnel plots.^34^

Paradoxically, in this study overly simplified data visualisations, pictorial data in the form of star ratings, were the least favoured cross-sectional data visualisation format. This finding also aligns with previous studies in other clinical settings, which have consistently shown that healthcare professionals tend to prefer basic summary data over simplified pictorial representations that omit statistical detail, even though pictorial data visualisations are often interpreted correctly.^22 35–37^ Evidence suggests that pictorial data are more appropriate for, and preferred by, consumer populations than healthcare staff.^38 39^ Despite this, the ease of interpretation of pictorials could still be leveraged in healthcare professional populations to enhance preferred formats. Integrating pictorial cues, such as icons or symbols, within conventional graphs (eg, icon-enhanced bar charts or annotated line graphs) can improve comprehension^40^; however, given staff dislike for pictorial formats, this approach should be applied carefully.

Across all visualisation formats in this study, at least one in five participants misinterpreted the data and only one participant answered all interpretation questions correctly. Low data literacy among healthcare staff is a well-documented issue^41–44^ that likely contributes to these findings.^20^ However, despite low levels of statistical training among Australian RAC staff,^34^ some participants in our study engaged with the data, highlighted its limitations, and showed familiarity with statistical concepts such as case-mix adjustment. This suggests that while formal training may be lacking, some staff possess foundational skills and are adapting to an increasingly data-driven environment in RAC.^11 16^ Open-ended responses further indicated that statistical methods, such as case-mix adjustment, and contextual information, such as facility size, were viewed as necessary by some participants to prompt action in data feedback cycles. To meet the information needs of staff with varying levels of data literacy in an increasingly data-driven environment, future quality reporting should aim for a balance between interpretability, simplicity and actionability. Given the high rates of misinterpretation observed, health systems and aged care providers must also ensure that the workforce is adequately trained and supported to engage meaningfully with increasingly complex performance data.^45^

### Implications for quality improvement practice

These findings suggest that in RAC settings, line graphs are the most appropriate format to support repeated-measure feedback cycles, while bar charts should be used to benchmark performance across facilities at a single time point or for early quality-improvement cycles, such as pre–post intervention comparisons.

RAC quality improvement activities should not assume that clear graphs alone ensure understanding, as all graph types in this study were misinterpreted by some participants. Health systems and RAC providers should actively and continuously build workforce data literacy through ongoing education and support, and present information in multiple complementary formats should promote accurate interpretation and practical use of care quality data.

### Strengths and limitations

The results of this study provide early insights into effective data visualisation formats for communicating performance to aged care staff, helping to address a critical evidence gap. However, several limitations should be noted. Importantly, this study captured perceived or intended action only; we did not measure observed real-world quality-improvement behaviour or downstream changes in care delivery, and findings should be interpreted accordingly. Further, the sample size was small despite extensive recruitment efforts and was not representative of the RAC workforce, limiting generalisability. For example, personal care workers comprised 30% of participants in this study, despite accounting for approximately 80% of the Australian RAC workforce.^46^ The small sample size and distribution across roles meant that descriptive statistics were the only appropriate analytic approach for this study; subgroup or inferential analyses would have been underpowered and potentially misleading. Low participation may reflect survey fatigue, as Australian RAC staff have been the focus of recent inquiries and sector reforms, and the perceived complexity of the survey, which may have resembled a statistics exam. It is also unknown how many eligible staff saw recruitment materials but chose not to participate as some recruitment efforts such as professional body forums and newsletters are open access. It is possible respondents in this survey may have had greater confidence in statistical skills or engaged frequently with quality improvement interventions. Finally, participants’ low preference for pictorial formats may have been influenced by the framing of the data in the survey, in which higher star ratings represented lower antipsychotic use, although similar star-rating systems (where low rate equates to high star rating) are widely used in healthcare and are therefore important to examine.

## Conclusion

Despite the small sample size, this study provides early, practice-relevant evidence to guide the design of data visualisations for medication-related quality improvement in RAC. Simple formats presenting summary statistics, bar charts for cross-sectional benchmarking and line graphs for longitudinal monitoring, were most consistently interpreted correctly, preferred by staff, and may therefore be more likely to support intended action within quality-improvement feedback cycles. Conversely, complex graphical data visualisations such as funnel plots and box plots may be less appropriate due to lower comprehension. The finding that at least one in five participants misinterpreted each visualisation type underscores that effective quality reporting in RAC depends not only on selecting appropriate formats but also on sustained investment in workforce data literacy to support accurate interpretation and action.

## Supplementary material

10.1136/bmjoq-2025-003897 online supplemental file 1

## Data Availability

Data are available upon reasonable request.
