# Supplementary material for: Optimising data visualisation formats for reporting medication-related care quality indicators: residential aged care staff preferences, interpretations and actions
Source: BMJ Open Qual. 2026 Sep 8;15(3):e003897. doi: 10.1136/bmjoq-2025-003897 (PMC13583859; doi:10.1136/bmjoq-2025-003897)
Supplement: Supplementary file 1 [file bmjoq-15-3-s001.docx]

Supplemental Material 1: RedCap survey

**Data visualisation and interpretation survey**

Heads up: This survey might give you flashbacks to a maths quiz! But don't worry, researchers at Macquarie University are only interested in finding out which graphs are the easiest to understand. We're not testing your maths skills-promise!

If you're a personal care worker, nurse, pharmacist, allied health professional, GP, manager, or policy maker in Australian residential aged care, we'd like your input! Your feedback will help us design graphs that are clear and useful for those working in aged care.

The survey is a one-time occurrence and takes about 15 minutes to complete.

If you finish the survey, you'll be entered into a draw to win a $50 gift voucher! We estimate that 1 in 10 participants will win. Prizes will be drawn on October 2nd, October 22nd, and November 18th-complete the survey early to get a chance in all three draws.

If you have any complaints regarding this survey, please email Macquarie University ethics: [ethics@mq.edu.au](mailto:ethics@mq.edu.au)

By clicking YES I agree to participate in this study and declare that:

- I have read the attached Participant Information Sheet, or someone has read it to me in a language that I understand.
- I understand the purposes, procedures and risks of the research described in the study. I am aware of any known or expected inconvenience, risk or discomfort and have informed the research team of any known health risks that may affect my participation.
- I have had an opportunity to ask questions and I am satisfied with the answers I have received.
- I freely choose to participate in this study, and I understand that I am free to withdraw at any time.
- If I decide to leave the study, I agree that the information collected about me up to the point when I withdraw may continue to be used by the researcher as it may be difficult to identify and remove my individual response.
- I understand that the research study is strictly confidential.

Yes No

**Just a few quick questions about you....**

In what sector are you employed? You may select more Residential aged care - Public (i.e. government than one option if relevant to you	funded)

 Residential aged care - Private

 Government agency/organisation Other

If your sector is not listed above, please specify

What is your current role/job classification? You may Assistant in Nursing/Personal Care select more than one option if relevant to you	Assistant/Personal Care Worker

 Enrolled Nurse Registered Nurse

 Nurse Practitioner

 Allied Health Practitioner General Practitioner

 Policy Advisor

 Residential/Facility Manager Care Manager

 Pharmacist Other

If your role is not listed above, please specify

How many years have you worked in the aged care	< 1 year

sector?	1-4 years

5-9 years

10 or more years

In your current role, do you encounter or use quality	Never

indicator data relating to medication management	Rarely (e.g., data relating to polypharmacy or antipsychotic	Sometimes medicine use etc.)? (optional)	Often

Always

Which of the following tasks do you do in your Provide governance, leadership and oversight day-to-day role? Please select all tasks that are Ensure there are adequate resources (e.g., relevant to you.	equipment, materials, appropriately trained staff

etc.) to support quality improvement activities Review performance and set targets to improve

quality of care

 Investigate and implement interventions that can improve care

 Personalise and discuss quality indicator data so it is relevant and meaningful to staff

 Collect, report and respond to quality indicators Check the reliability and accuracy of collected

quality indicator data

 Report quality indicator information to residents, and their families

 Investigate if there are changes in the care needs of residents (e.g., more complex needs), and make appropriate adaptations to improve care

 Partner with other organisations or facilities to share information to improve care

 Other (please specify)

What additional quality improvement tasks do you do in your day-to-day role?

**Hang in there! This is the longest part, but once you're through you'll have just one short section to go.**

Case study #1: Antipsychotic use in residential aged care facilities

Antipsychotic use is defined as the percentage of residents in each facility who received an antipsychotic during a one-week data collection period.

Data format 1

Figure 1 illustrates the use of antipsychotic medicines across 15 individual facilities, arranged in order from the highest use to the lowest. The dashed line shows the average (mean) use for all facilities.

What is the average antipsychotic use among these	28%

facilities?	18%

20%

15%

Not sure

Compared to all other facilities, antipsychotic use	Above the average

for Facility A is... (please complete the sentence)	Consistent with the average Below the average

Not sure

The rate of antipsychotic use reported for Facility May indicate inappropriate use of medicines. A... (please complete the sentence)	Further investigation/action may be needed

Indicates that no action is required. Not sure

Other

If other, please specify

How easy is it to interpret this figure?	Very difficult Difficult Neutral

Easy Very easy

Does this graph provide enough information for you to take action to improve antipsychotic use in

Yes No

residential aged care facilities?	Not sure

If yes, what action would you take?

If no, what additional information do you need?

Data format 2

Table 1 below summarises the percentage of residents who received an antipsychotic medicine, across 15 individual facilities. Facilities with antipsychotic use above the mean (average) are shown in orange font, and those with antipsychotic use below the mean (average) are shown in blue font.

Note: The confidence interval (95% CI) for each facility is calculated using statistical methods and indicates the range of plausible average (mean) values for that facility.

Which facility has the highest percentage of residents	Facility F who received an antipsychotic medicine?	Facility H

Facility I Not sure

The antipsychotic use reported for Facility F... May indicate inappropriate use of medicines. (please complete the sentence)	Further investigation/action may be needed

Indicates that no action is required. Not sure

Other

If other, please specify

How easy is it to interpret this table?	Very difficult Difficult Neutral

Easy Very easy

Does this table provide enough information for you to take action to improve antipsychotic use in

Yes No

residential aged care facilities?	Not sure

If yes, what action would you take?

If no, what additional information do you need?

Data format 3

The funnel plot below illustrates the percentage of residents at each facility who received an antipsychotic medicine. The average (mean) across all facilities is shown as a dashed horizontal line. Facilities with percentages between the two orange lines are within the average range. Facilities with percentages outside these lines have higher (above the upper line) or lower (below the lower line) than average use.

What is the average antipsychotic use across these	8.5%

facilities?	24.0%

10.0%

14.5%

Not sure

How many facilities have antipsychotic use above the	2 facilities orange warning limit?	1 facility

6 facilities All facilities No facilities Not sure

Compared to all other facilities, antipsychotic use	Above the average range

for Facility A is... (please complete sentence)	Within the average range Below the average range Not sure

The rate of antipsychotic use reported for Facility May indicate inappropriate use of medicines. A... (please complete sentence)	Further investigation/action may be needed

Indicates that no action is required. Not sure

Other

If other, please specify

How easy is it to interpret this figure?	Very difficult Difficult Neutral

Easy Very easy

Does this graph provide enough information for you to	Yes take action to improve antipsychotic use in	No

residential aged care facilities?	Not sure

If yes, what action would you take?

If no, what additional information do you need?

Data format 4

The caterpillar plot below shows antipsychotic use in 15 facilities. Dots indicate the percentage for each facility and the vertical lines (confidence intervals) indicate a range of plausible values for each facility. The horizontal line indicates the average (mean) across all facilities. If the confidence interval does not cross the horizontal line, the facility has higher (orange) or lower (blue) than average antipsychotic use.

What is the average antipsychotic use across these	14.5%

facilities?	10.0%

23.0%

30.5%

Not sure

Compared to all other facilities, antipsychotic use	Above the average range

for Facility H is... (please complete sentence)	Within the average range Below the average range Not sure

The rate of antipsychotic use reported for Facility	May indicate inappropriate use of medicines. H... (please complete sentence)	Further investigation/action may be needed

Indicates that no action is required. Not sure

Other

If other, please specify

How easy is it to interpret this figure?	Very difficult Difficult Neutral

Easy Very easy

Does this graph provide enough information for you to	Yes take action to improve antipsychotic use in	No

residential aged care facilities?	Not sure

If yes, what action would you take?

If no, what additional information do you need?

Data format 5

The table below represents antipsychotic use at 15 facilities as a performance rating. Three stars indicate facilities performing within the average range, 1 or 2 stars indicate facilities with higher antipsychotic, and 4 or 5 stars indicate facilities with lower antipsychotic use.

How many facilities are performing within the average	4 facilities range?	1 facility

9 facilities All facilities Not sure

Compared to all other facilities, Facility O is...	Performing better than average (please complete sentence), for antipsychotic use	Within the average range

Performing worse than average Not sure

Antipsychotic use reported for Facility O... (please	May indicate inappropriate use of medicines. complete sentence)	Further investigation/action may be needed

Indicates that no action is required. Not sure

Other

If other, please specify

How easy is it to interpret this table?	Very difficult Difficult Neutral

Easy Very easy

Does this table provide enough information for you to	Yes

take action to improve antipsychotic use in	No

residential aged care facilities?	Not sure

If yes, what action would you take?

If no, what additional information do you need?

Data format 1

Below is a summary of the data presentation formats you have seen in this case study.

Data format 2

Data format 3

Data format 4

Data format 5

Which data presentation format did you prefer the	Data format 1

most?	Data format 2

Data format 3

Data format 4

Data format 5

Which data presentation format did you prefer the	Data format 1

least?	Data format 2

Data format 3

Data format 4

Data format 5

**You're almost there! This is the final stretch, and it's way shorter than the last part. Just a few more clicks, and you'll be in the running for that $50 gift voucher!**

Case study #2: Polypharmacy in residential aged care facilities

Polypharmacy is defined as the percentage of residents in each facility who were prescribed 9 or more medicines on the data collection day.

Data format 1

The figure below illustrates polypharmacy among 15 facilities from January 2023 to December 2023. A time series of boxplots allows comparison of the median (the wider solid line inside the box) and the variation (represented by the height of the box and vertical lines) over time. The coloured dots represent facilities which are "outliers".

What is the average (median) polypharmacy use across	25% all facilities in June 2023?	40%

56%

45%

Not sure

Are there any outlier facilities in June 2023?	No

Yes, 1 facility

Yes, 2 facilities

Yes, 3 facilities Not sure

Please indicate in which months Facility N has	Jan

polypharmacy use that is outside expected levels of	Feb

variation (select as many as relevant)	Mar Apr May Jun Jul Aug Sep Oct Nov Dec

Not sure

The polypharmacy use reported for Facility F in	May indicate inappropriate use of medicines. December 2023... (please complete sentence)	Further investigation/action may be needed

Indicates no action is required Not sure

Other

If other, please specify

How easy is it to interpret this figure?	Very difficult Difficult Neutral

Easy Very easy

Does this graph provide enough information for you to	Yes take action to improve polypharmacy in residential	No

aged care facilities?	Not sure

If no, what additional information do you need?

If yes, what action would you take?

Data format 2

The figure below illustrates monthly polypharmacy among facilities from January 2023 to December 2023. The blue line indicates polypharmacy for Facility A across the 12-month period, and the grey line represents the average (mean) polypharmacy of all other facilities. Shading indicates the 95% confidence interval which shows the range of plausible values for each average (mean) value.

| What is the average polypharmacy across all other | 45% |
| --- | --- |
| facilities in April 2023? | 50% |
|  | 41% |
|  | 30% |
|  | Not sure |

Compared to all other facilities, polypharmacy for	Above average

Facility A in February 2023 is... (please complete	Within the average range

sentence)	Below average

Not sure

Polypharmacy reported for Facility A in July 2023...	May indicate inappropriate use of medicines. (please complete sentence)	Further investigation/action may be needed

Indicates no action is required Not sure

Other

If other, please specify

How easy is it to interpret this figure?	Very difficult Difficult Neutral

Easy Very easy

Does this graph provide enough information for you to	Yes take action to improve polypharmacy in residential	No

aged care facilities?	Not sure

If yes, what action would you take?

If no, what additional information do you need?

CS2 Data format 1

CS2 Data format 2

Which of the following data presentation formats do you prefer?

Don't forget to hit submit to make sure you go in the draw for a $50 gift voucher!

**Supplemental Table 1: The professional roles of participants who completed the survey and those who did not complete the survey**

| Role | Completed the survey | Did not complete the survey | Total |
| --- | --- | --- | --- |
| Assistant in Nursing | 12 | 19 | 31 |
| Registered Nurse | 10 | 5 | 15 |
| Nurse Practitioner | 0 | 1 | 1 |
| Allied Health Practitioner | 2 | 1 | 3 |
| General Practitioner | 1 | 0 | 1 |
| Policy Advisor | 2 | 1 | 3 |
| Residential/Facility | 1 | 2 | 3 |
| Care Manager | 3 | 1 | 4 |
| Pharmacist | 6 | 0 | 6 |
| Other | 5 | 8 | 13 |
| Total | 42 | 38 | 80 |
| Fisher's exact =0.07 | | | |

**Supplemental Table 2: Experience with quality indicator data in participants who completed the survey and those who did not complete the survey**

| In your current role, do you encounter or use quality indicator data? | Completed the survey | Did not complete the survey | Total |
| --- | --- | --- | --- |
| Never | 5 | 9 | 14 |
| Rarely | 2 | 5 | 7 |
| Sometimes | 4 | 6 | 10 |
| Often | 10 | 6 | 16 |
| Always | 18 | 9 | 27 |
| Total | 39 | 35 | 74 |
| Fisher's exact =0.163 | | | |

**Supplemental Table 3: Years of experience of people who completed the survey and those who did not complete the survey**

| How many years have you worked in the residential aged care sector? | Completed the survey | Did not complete the survey | Total |
| --- | --- | --- | --- |
| < 1 year | 8 | 6 | 14 |
| 1-4 years | 10 | 14 | 24 |
| 5-9 years | 9 | 7 | 16 |
| 10 or more years | 15 | 11 | 26 |
| Total | 42 | 38 | 80 |
| Fisher's exact= 0.668 | | | |

**Supplemental Table 4. Coding of opened ended responses**

| Themes | Bar chart | Table | Funnel chart | Caterpillar plot | Star rating | Box plot | Line graph |
| --- | --- | --- | --- | --- | --- | --- | --- |
| **If this graph type provides you with enough information, what action would you take?** | | | | | | | |
| I would investigate the reason for medication use at the resident and/or facility level | 8 | 3 | 4 | 2 | 2 | 0 | 2 |
| I would organise or perform a medication review and/or work with the facility to implement strategies to improve medication use | 3 | 3 | 3 | 3 | 3 | 3 | 4 |
| I believe that this graph type is easy to read and/or contains complete information | 1 | 2 | 2 | 2 | 1 | 0 | 2 |
| I would continue to monitor medication use | 0 | 0 | 0 | 0 | 0 | 0 | 1 |
| Number of respondents | 12 | 8 | 9 | 7 | 7 | 3 | 11 |
| **If this data visualisation does not provide you with enough information, what additional information do you need?** | | | | | | | |
| I need more contextual information e.g., facility size and case mix of facilities | 7 | 4 | 5 | 4 | 5 | 2 | 0 |
| I needed a clearer data visualisation, this is unclear/confusing | 0 | 0 | 1 | 2 | 1 | 4 | 0 |
| Number of respondents | 7 | 4 | 6 | 6 | 6 | 6 | 0 |
